# Normal vs. Malignant hematopoiesis: the complexity of acute leukemia through systems biology

**DOI:** 10.3389/fgene.2015.00290

**Published:** 2015-09-11

**Authors:** Jennifer Enciso, Luis Mendoza, Rosana Pelayo

**Affiliations:** ^1^Oncology Research Unit, Mexican Institute for Social SecurityMexico City, Mexico; ^2^Biochemistry Sciences Program, Universidad Nacional Autónoma de MéxicoMexico City, Mexico; ^3^Departamento de Biología Molecular y Biotecnología, Instituto de Investigaciones Biomédicas, Universidad Nacional Autónoma de MéxicoMexico City, Mexico

**Keywords:** acute leukemia, early hematopoiesis, bone marrow, mathematical modeling, regulatory networks, systems biology

## The early stages of malignant hematopoiesis: A multi-cellular, multi-compartment and multi-factorial challenging study model

Development of normal hematopoietic cells is an ordered multi-step process, tightly regulated by a complex network of intrinsic factors and microenvironmental cues that control cell fate decisions within the bone marrow (BM) (Pelayo et al., 2012; Purizaca et al., 2012; Boulais and Frenette, 2015). During malignant hematological disorders, including acute leukemias (AL), the uncontrolled differentiation of precursors of the lymphoid or myeloid series sustains tumor growth at the expense of normal blood cell production. Moreover, selection and dominance among leukemic clones occur while competing for niche resources and creating abnormal BM microenvironments that co-participate in the pathobiology of the disease (Colmone et al., 2008; Ayala et al., 2009; Purizaca et al., 2012; Kim et al., 2015; Vilchis-Ordoñez et al., 2015). Thus, due to the complexity and health impact of AL (Gupta et al., 2014), new strategies to better predict cell population dynamics according to genetics, microenvironmental and clinical heterogeneous contexts may contribute to understand its pathobiology and to guide strategies for decreasing overall mortality.

Mathematical modeling has emerged as a powerful tool in biomedical and health research because it enables the simulation of complex biological systems and the efficient generation of testable hypotheses. In recent years, leukemic cell dynamics has been addressed from the novel view of systems biology, resulting in helpful stochastic and deterministic models and providing clearer understanding of the disease by simplification of malignant clonal evolution processes (Vesely et al., 2011; Amir et al., 2013; Paguirigan et al., 2015). However, models fitted to experimental data must strike a balance between simplicity and reality, so that they can bring insights into clinical scenarios.

Here we discuss the importance and challenges of incorporating the BM microenvironment into AL modeling, as a key element that will control the interplay between cell populations and the selective pressure leading to leukemic or normal hematopoiesis progression. By developing integrative tools that better mimic and predict the behavior of heterogeneous and polyclonal cells in the context of abnormal microenvironments within leukemic bone marrow, we may learn about crucial mechanisms co-participating in the etiology and progression of the disease.

## Normal vs. leukemic clones: Systems biology in the study of acute leukemia complexity

Continuous dynamic modeling with differential equations (DEs) has been the most popular systems biology tool for the study of normal and leukemic hematopoiesis. This type of modeling is useful for the time evolving non-linear competition between normal and leukemic cell populations, considering multiple compartments to simulate different maturation stages or multiclonal behavior (Catlin et al., 2005; Stiehl and Marciniak-Czochra, 2012; MacLean et al., 2013; Stiehl et al., 2014).

Of special interest, theoretical data suggests the existence of an initial “steady state” before the disease development, when co-existence of normal hematopoiesis with a limited number of pre-leukemic cells controls leukemia installation (Rubinow and Lebowitz, 1976; Stiehl and Marciniak-Czochra, 2012; Swaminathan et al., 2015). A sudden change in the homeostatic parameters may induce leukemic cell expansion leading to a progressive decrease of normal hematopoiesis, while perturbation of initial homeostatic state endows malignant cells with self-renewal and proliferation. Accordingly, the model by Rubinow and Lebowitz's on competition advantage of leukemia cells proposed a higher value of their equilibrium number that refers to the maximum population size that can be supported within the niche. If the stop-expansion signal for malignant progenitors is not delivered before the equilibrium number is reached, a signal activating the slow-down of normal cells promotes the expansion of the leukemic population. High equilibrium numbers in leukemic compartments could be biologically interpreted as independence from the microenvironment, unbalanced proliferation/apoptosis rates, and further accumulation of blasts.

Using a stochastic model to simulate stem cell decisions, Abkowitz and colleagues have analyzed the behavior of individual components (HSC) acting collectively within a dynamical complex context (clonal diversity plus heterogeneous surrounding microenvironment). By tracking HSC replication, the expansion of the hematopoietic system was apparent from birth to adolescence, when steady-state levels are reached. Stochastic modeling of replication kinetics has shown to be useful to predict cell rebounding upon hematopoietic transplantation or under emerging conditions (Catlin et al., 2005, 2011). In contrast, agent-based deterministic modeling of HSC organization in health and hierarchical-related diseases, like chronic myeloid leukemia, are powerful for simulating additional heterogeneity scenarios to be considered, i.e., aging, HSC-niche interaction and therapy outcomes (Glauche et al., 2011). Unlike CML, AL cells show apparent dependence on their own “leukemic niche” (Veiga et al., 2006; Colmone et al., 2008; Basak et al., 2010; Jacamo et al., 2014). Recent models suggest additional feedback mechanisms assuming both, the leukemic and normal cell interdependence on the same growth factors (Stiehl et al., 2014).

In addition to the normal vs. leukemic competition, increasing evidence of genetic diversity supports the multiclonal evolution of AL (Choi et al., 2007; van Delft et al., 2011; Amir et al., 2013). Strikingly, rather than as a consequence of new acquired mutations, relapse could be explained as a deterministic clonal selection where high proliferative cells are eliminated by chemotherapy, while distinct slow-cycling or self-renewing cells stay protected and may re-emerge when the competing clones (leukemic high-proliferating cells) and their negative feedback (normal hematopoietic cells) have been eliminated. Similar to deterministic models of chemotherapy-dependent clonal selection, the stochastic modeling by Kimmel and Corey drives to the conclusion on the co-existence of distinct clones and the extremely broad heterogeneity of cancer cells. However, the stochastic acquisition of mutations may provide theoretical evidence of the parallel evolving clones with unique proliferative potentials, and represent a suitable model for chronic chemotherapy-induced transition to secondary malignancy (Kimmel and Corey, 2013). Despite the fact that linear mutation structures can simplify the population dynamics, it is necessary to consider proliferation heterogeneity. Interestingly, the acquisition of *de novo* mutations is more probable during long treatment schemes (Lindsley et al., 2015).

Technological advances in RT-PCR, RNA-seq and mass cytometry methods for single cell analysis are providing highly specific clusterization of cell populations that allow the identification of experimentally unseen cell transition stages from the earliest steps of differentiation (Marco et al., 2014; Moignard et al., 2015). With new experimental models and molecular research progress, parameters and assumptions considered for the development of mathematical models, evolve to a more complex understanding of leukemogenesis. The view of two or more hematopoietic populations competing within compartments, plus the resulting regulation among compartments from the isolated feedback loops is too simplistic. Therefore, it is becoming of substantial importance to take into account additional intercellular interactions, including those with non-hematopoietic neighboring cells within the BM niches.

## Modeling the interplay between leukemia cells and the tumor microenvironment

Tumor-microenvironment interplay is essential for the protection and progression of malignant cells, where a number of interactions mediated by integrins, cytokines and chemokines, extracellular matrix (ECM) proteins, and other molecules produced and expressed by niche cellular elements, may dictate the final fate decision (Raaijmakers, 2011). The recent multi-compartment model by Gerdes for T-cell lymphoma/leukemia, suggests that premalignant cells can get established in any available permissive niche, compensating their low affinity for specific interactions with an increased efficiency for resource utilization when compared to normal clones (Gerdes et al., 2013).

Closer to this multi-component interaction outlook has been the development of generic-cancer cell-automata models. This type of discrete modeling makes the evaluation of homogeneous or heterogeneous cell populations in a grid where every cell has a defined state and neighborhood possible. Strikingly, cell-automata modeling concede single-cell resolution and had become a very promising tool for the study of tissue development and tumors, including microenvironmental factors like ECM density and oxygen diffusion that control tumor size (Chen et al., 2014; Scott et al., 2014).

In spite of the power of these approaches, it is clear that the feedback existing between the BM microenvironmental components and the malignant cell decisions operates at a molecular level regulating intracellular pathways. How could we mimic the complexity at cellular population and molecular levels at the same time? How could we address the multi-cellular system within systems complexity?

## Simulation of one-cell molecular network models with multi-cellular methods

**Figure 1:**
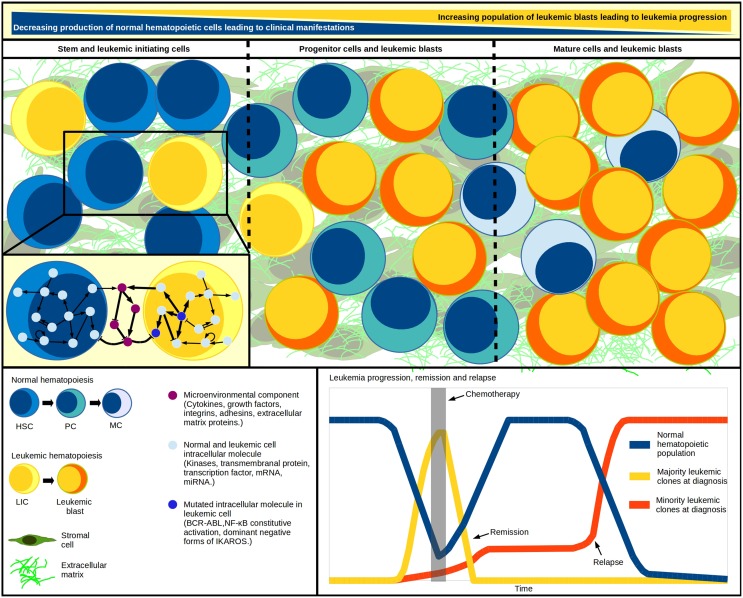
**Systems within a system**. Leukemic initiation and progression is a tightly regulated competitive process, where at least three systems must work together: the normal hematopoietic differentiation, the leukemic cell production, and the hematopoietic microenvironment where malignant and normal cells competition takes place. As blast population increases, the normal cell abundance decreases. Cell population changes have been modeled through differential equations in multi-compartment continuous modeling, strategy that allows the representation of hematopoietic hierarchy with the assignation of different kinetic parameters values for cells within each compartment. The model mimics *in vivo* fundamental properties like quiescence in the stem-cells compartment and increasing proliferation in developing cells. Additional to regulatory feedback between normal and malignant hematopoietic populations, an abnormal microenvironment may play a crucial cooperating role in the inverse leukemic/normal relationship by disrupting the HSC-niche communication. The genetic diversity within the various leukemia-initiating cells and tumor cells highlights the multiclonal complexity of the disease, and suggests the existence of minor malignant clones—undetected at diagnosis—that become apparent upon chemotherapy and drive individuals to relapse. HSC, hematopoietic stem cell; HPC, hematopoietic progenitor cell; PC, precursor cell; MC, mature cell; LIC, leukemia initiating cell.

Knowledge about the hematopoietic system has been benefited from the development of regulatory networks for early HSC differentiation, T lymphocyte development, plasticity and signaling, among others (Albert and Wang, 2009; Naldi et al., 2010; Martínez-Sosa and Mendoza, 2013; Tian and Smith-Miles, 2014). Considering that every computational simulation with a specific initial state of an intracellular network represents a single cell dynamic profile, to simulate a multi-cellular process we must simultaneously simulate as many networks as cells within the system (Wu et al., 2009). Accordingly, Mendoza proposed a virtual culture of Th cells that simulate differentiation of naive CD4+ T cells to Th1, Th2, Th17, and Treg subsets. In this model, each cell phenotype is defined by molecular patterns of activation, while the input for each virtual cell at any time-step proceeds from the intercellular communication (Mendoza, 2013). More importantly, the dynamics of a given regulatory network respond to the concentration of regulatory cytokines produced by the cell itself and to neighbor signal intensities. Thus, applying tools like virtual cultures to malignant hematopoiesis may help to understand blast accumulation or the intercommunication between leukemia-initiating cells and an abnormal BM microenvironment (Figure 1). The recent demonstration of pro-inflammatory cytokines produced by ALL cells suggests that this condition may promote their own survival and account for the exhaustion of normal progenitor cells (Vilchis-Ordoñez et al., 2015). The pathological consequences of a pro-inflammatory microenvironment can be resumed in three potential principles: (a) leukemic cells showing aberrant expression of cytokines that perturb normal hematopoiesis, (b) mutated stromal cells favoring a permissive microenvironment for leukemia initiation, progression, and maintenance (Shalapour et al., 2010), or (c) normal hematopoietic cells responding to biological stress due to blast overcrowding by activating pro-inflammatory pathways. These three scenarios might act independently or sinergistically by means of positive feedback.

To solve this, hybrid models are also mathematical tools with great potential to model microenvironment-dependent systems, allowing the scaling to tridimensional modeling and the consideration of discrete decisions on cell processes like migration and proliferation (Anderson, 2005; Scott et al., 2014). Although these dedicated models have considered microenvironmental factors for solid tumor progression, they still miss the direct feedback existing between extracellular factors and the intra-cellular signaling pathways that regulate cell fate decisions. Of note, an intracellular view would allow modeling of constitutive or null activation of specific pathway mediators and analyzing the putative consequent effects on disease dynamics. Virtual cultures make this possible, but the very high computational requirements when modeling excessive number of cells may represent by now a weakness of the strategy.

For any of the discussed modeling approaches, the importance of a rigorous experimental validation of mathematical modeling for complex processes is high and has been limited by the experimental systems that are conventionally used to study human leukemogenesis. The combination of single-cell sequencing, 3-D organoid-like cultures and xenotransplantation would provide new information for malignant vs. normal cell discrimination and cell population dynamics within more natural microenvironmental structures. Furthermore, a proper validation of current and future investigations from the view of systems biology will benefit from longitudinal, prospective clinical studies.

To this extent, the use of “edge-technology” *in silico* strategies for multi-cellular (leukemic, hematopoietic, and stromal components), multi-compartment (differentiation stages), and agent-based (individual cells network) modeling of leukemia pathobiology is a promising tool for the study of feedback pathways in the searching of auxiliary strategies for leukemia treatment, normal hematopoiesis rebounding, and relapse delay. The construction of novel “systems within a system” integrative theoretical models (Figure 1) that better mimic and predict the behavior of the disease may transform our vision of malignant hematopoiesis and provide helpful platforms for new testable hypotheses.

## Author contributions

JE: Analysis of published data, discussion of the topic-related information, drafting, and writing the paper. LM and RP: Conception and design of the Opinion Article, analysis of published data, discussion of the related information, drafting, and writing the paper. Critical review of the intellectual content.

### Conflict of interest statement

The authors declare that the research was conducted in the absence of any commercial or financial relationships that could be construed as a potential conflict of interest.
